# Seismic landslide susceptibility assessment using principal component analysis and support vector machine

**DOI:** 10.1038/s41598-023-48196-0

**Published:** 2024-02-14

**Authors:** Ziyao Xu, Ailan Che, Hanxu Zhou

**Affiliations:** School of Naval Architecture, Ocean and Civil Engineering, Shanghai Jiao Tong University, 800 Dongchuan Road, Shanghai, 200240 China

**Keywords:** Ecology, Natural hazards

## Abstract

Seismic landslides are dangerous natural hazards that can cause immense damage to human lives and property. Susceptibility assessment of earthquake-triggered landslides provides the scientific basis and theoretical foundation for disaster emergency management in engineering projects. However, landslide susceptibility assessment requires a massive amount of historical landslide data. Evidence of past landslide activities may be lost due to changes in geographical conditions and human factors over time. The lack of landslide data poses difficulties in assessing landslide susceptibility. The aim of this study is to establish a generalized seismic landslide susceptibility assessment model for applying it to the Dayong highway in the Chenghai area, where earthquakes occur frequently but with a lack of landslide data. The landslide data used comes from the 2014 Ludian Ms (Surface wave magnitude) 6.5 earthquake in a region with geographical conditions similar to those in the Chenghai area. The influencing factors considered include elevation, slope, slope aspect, distance to streams, distance to faults, geology, terrain wetness index, normalized difference vegetation index, epicenter distance and peak ground acceleration. The frequency ratio method is used to eliminate influencing factors with poor statistical dispersion of landslides. Principal component analysis (PCA) is utilized to reduce the dimensionality of landslide conditioning factors and to improve the transferability of the assessment model to different regions. A support vector machine model is used to establish the susceptibility assessment model. The results show that the accuracy of the PCA–SVM model reaches 93.6%. The landslide susceptibility of the Chenghai area is classified into 5 classes, with the “Very high” landslide susceptibility class accounting for 0.63%. The 13-km section in the middle of the Dayong highway, which accounts for 8.9%, is identified as the high-risk area most obviously impacted by seismic landslides. This study provides a new approach for seismic landslide susceptibility assessment in areas lacking in landslide inventory data.

## Introduction

Landslides represent one of the most destructive and frequent natural hazard phenomena reported worldwide^1^. It creates a significant constraint in the pace of economic development due to the disruption of infrastructures and blockades of transportation facilities^2^. Landslide susceptibility assessment is an effective approach which has been widely used to restrict and affect project planning to reduce damage from landslides to public property, infrastructure, and people’s lives^3–5^.

A wide range of qualitative and quantitative approaches have been used for landslide susceptibility assessment^6^. The approach mainly includes assessments based on expert experience and sophisticated mathematical methods^7^. The assessments based on expert experience contain discriminant analysis^8^, analytical hierarchy process^9,10^. Those methods based on experts’ knowledge and experience, ratings by different experts often lead to different assessment results^11^. The main mathematical and statistical methods include the logistic regression model and the weight of evidence method^12^, and the Index of Entropy (IoE) and Dempster–Shafer (DS) models. Those methods based on the available landslide data, assess various classifications of landslide influence factors^13^, and figure out the correlation between landslide susceptibility and influence factors. However, neither of them is suitable for dealing with data imbalance and nonlinearity problems.

With the development of computing power and geospatial data, many machine learning methods such as support vector machine (SVM), logistic regression^14^, random forester (RF), boosted regression tree (BRT), artificial neural network (ANN)^15^, recurrent neural networks (RNNs)^16^ and convolutional neural network (CNN)^17,18^ have been developed. The machine learning models performed better on nonlinear problems. In particular, SVM becomes popular in the landslide susceptibility assessment due to its characteristics like small number of samples, nonlinearity, high dimension, and fast learning capacity^19,20^. Based on the statistical learning theory, SVM aims to find a linear hyperplane in the feature space which could separate the positive and negative samples with the maximum margin. Therefore, the SVM is widely used in identification of landslides.

In the past decades, many studies have been conducted to assess the susceptibility of landslide using SVM or other machine learning methods^21^. The performance of the SVM and other different machine learning algorithms are compared in assessment of earthquake-triggered landslide susceptibility^22,23^. Zhou^24^ applied the SVM, ANN and a multivariate statistical model, the logistic regression for landslide susceptibility modeling. Huang^25^ proposed a hybrid modeling approach using support vector machines and random subspace. Tested it in the Wuning area to produce a landslide susceptibility map. Razavi^26^ employed adaptive neuro-fuzzy inference system in an ensemble with the ant colony optimization and differential evolution algorithms for the landslide susceptibility map of the Fahliyan sub-basin. In addition, many studies assessed the direct losses resulting from landslides on engineering such as highway^27–30^. Yin^31^ combined the PCA and SVM model for the susceptibility mapping and zoning of highway landslide disasters in China. According to the aforementioned methods, most researches on landslides along the highway based on historical landslides samples in the area.

However, previous studies on machine learning and SVM mostly focused on the accuracy comparison. The limitation of these research is that the landslide susceptibility models are highly dependent on the number of landslide samples, resulting in poor performance confronting the data deficiency. Due to unique topographical features of different areas, it is difficult to apply the landslide susceptibility model trained by landslide data from one area to another landslide data deficiency area. Therefore, it is crucial to improve the robustness of the assessment model through data processing method and to apply it to the area with a lack of landslide data but frequent earthquakes.

This study focuses on the application of the seismic landslide susceptibility assessment in the area where earthquakes occur frequently and there is an absence of landslide data. Dayong Highway located in Chenghai region is a representative area to be selected as the research background which is affected by Chenghai fault zone with earthquakes occurred frequently but landslide data deficiency. The seismic landslide susceptibility model is established based on the 716 landslides caused by Ludian earthquake and 10 influenced factors. PCA is adopted for reducing complexity of input variables and making the influence factors dimensionless. The robustness of model is further increased for applying in Dayong Highway of the Chenghai earthquake-prone area with geological conditions similar to Ludian area. We use Gutenberg-Richter model and Dieterich model to assume an earthquake in Chenghai area based on historical earthquakes. Finally, the study ends up with the assessing the landslide risk class of Dayong highway in Chenghai region based on the Inverse Distance Weight (IDW) method.

## Study area

**Figure 1:**
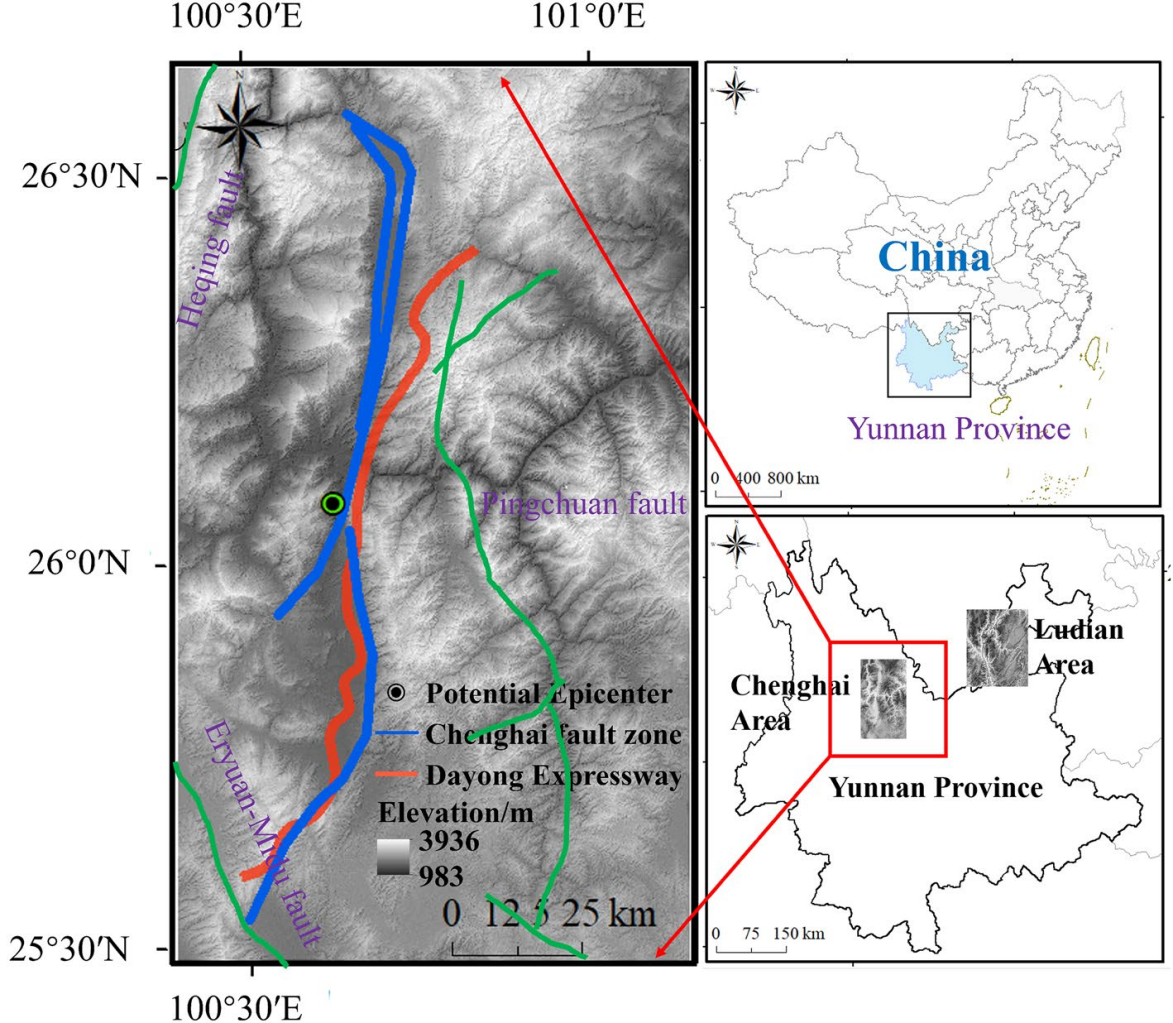
Highway and Chenghai fault zone of study area. (Credit: 1,2,5,6,11,12, ArcGIS10, URL: https://gisserver.domain.com:6443/arcgis/manager).

Chenghai area is located in the northwest of Yunnan Province. Because of the relative movement of the Eurasian and Indian Plate, a series of N–S, N–W and N–E trending faults formed the diamond-shaped Dali fault system^32^. This region is affected by many active faults, with frequent occurrence of strong earthquakes, which poses very serious risks of earthquakes triggering geological disasters. The geographical location of Chenghai area is shown in Fig. 1.

**Figure 2:**
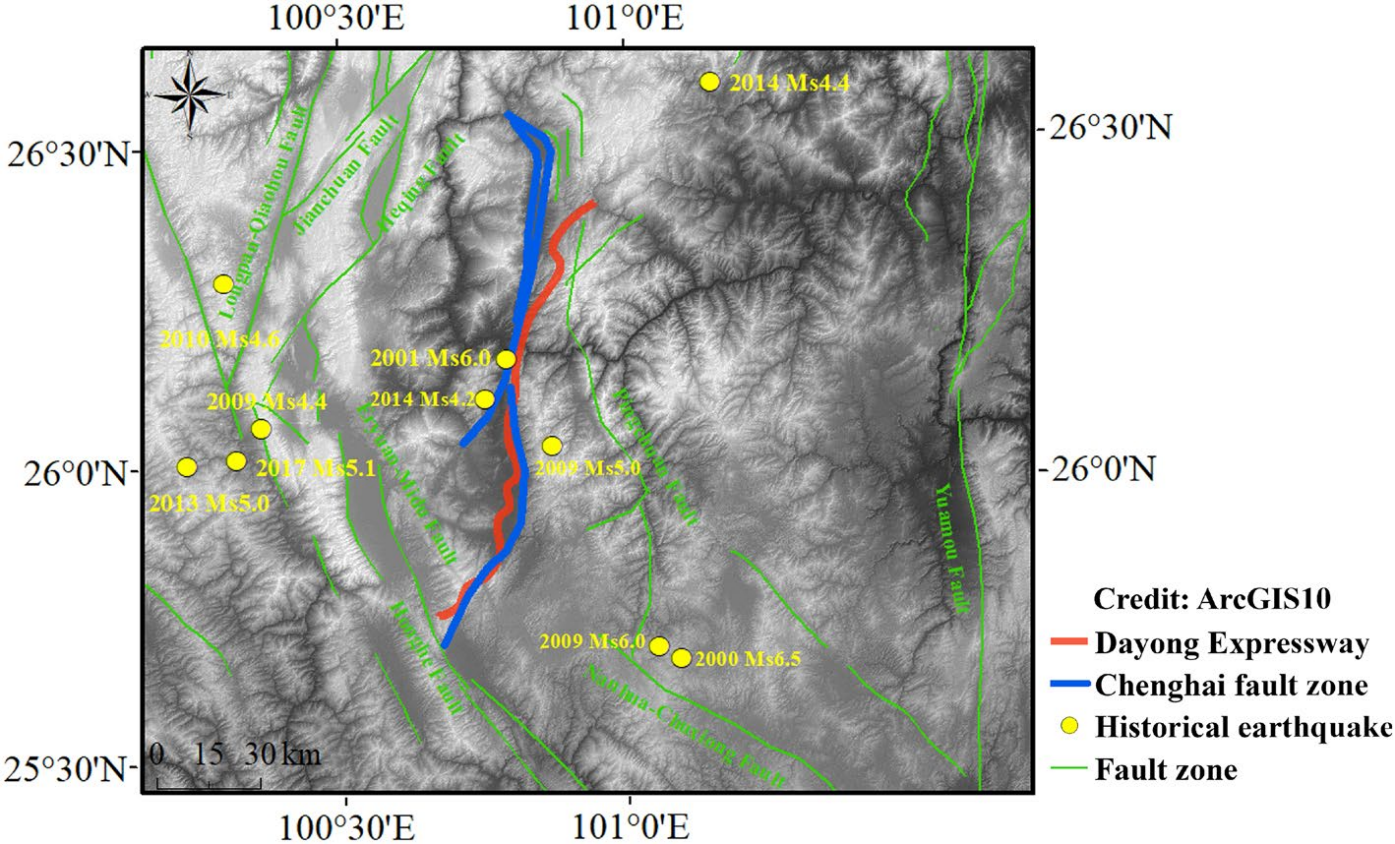
Regional seismic activity of Chenghai area. (Credit: 1,2,5,6,11,12, ArcGIS10, URL: https://gisserver.domain.com:6443/arcgis/manager).

Chenghai fault zone lies to the Midwest of the diamond-shaped Dali fault system and is surrounded by three regional active faults, including Heqing fault (W), Eryuan-Midu fault (SW), Pingchuan faults (E) and Jianchuan fault (NW). The dense distribution of faults and special geological conditions lead to the frequent seismic activities in this area. Most of the earthquakes occurred are closely related to these fault structures. From 1970 to 2015, there were 575 earthquakes above magnitude 3 in Chenghai region, among which 3 were strong earthquakes above magnitude 4. Such as the Yaoan Ms6.5, 2009, Yongsheng Ms4.9, 2019 and Ninglang Ms5.7, 2012. These earthquakes were characterized by frequent occurrences and small magnitudes, they mostly occurred near the fault, thereby indicating that the tectonic activity in this area is vigorous^33^. The fault with the largest effect on the Dayong Highway is the Chenghai fault. Dayong highway in the east of Chenghai fault zone. The strike of the line is roughly parallel 60 km to the Chenghai fault zone, and most of the lines passes through this zone. In 1915, the activity of the Chenghai fault zone was one of the causes for the occurrence of the 7.8 magnitude Yongsheng earthquake. Figure 2 describes the regional earthquakes in Chenghai area.

The Dayong Expressway is located in the Chenghai area. It is a bidirectional four-lane highway with a total length of 126 km. The areas surrounding this expressway are affected by geological structures and historical earthquakes, with the presence of many adverse slopes. Through the analysis of historical earthquakes in the Chenghai region, it is believed that the environment around the Dayong Expressway is influenced by the Chenghai fault structure. Earthquake activities are very likely to occur in the future, which would affect the safety of the expressway.

## Methodology

**Figure 3:**
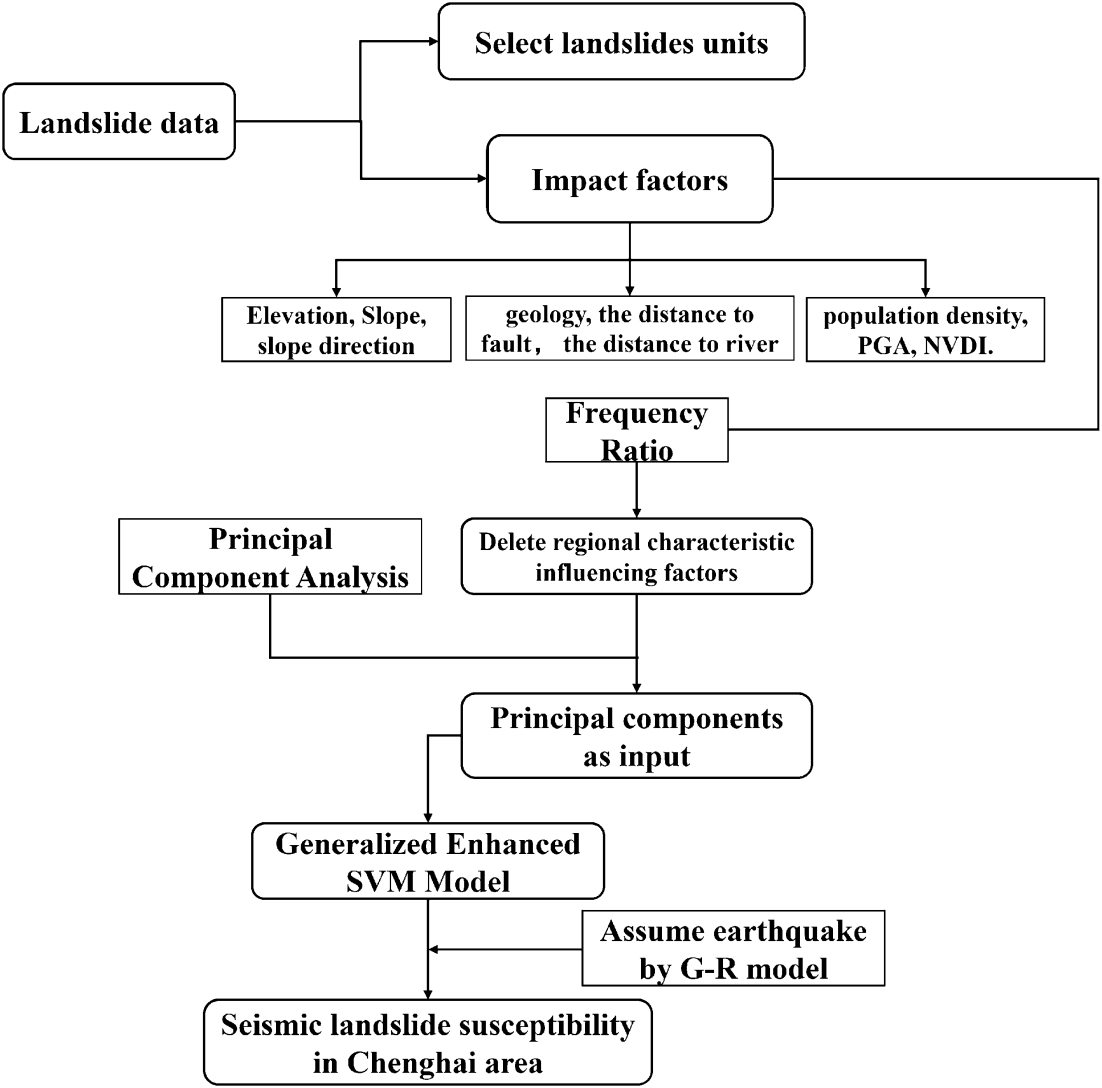
Flow chart of landslide susceptibility assessment.

In this study, two methods—Principal Component Analysis (PCA) and a Support Vector Machine (SVM) model—are combined in a GIS environment for seismic landslide susceptibility assessment. As shown in Fig. 3, the entire process is divided into two parts: data collection and model application. First, seismic landslide influencing factors and landslide samples from the Ludian earthquake are selected. Then, a seismic landslide susceptibility model is established using the SVM based on Ludian seismic landslide data. Next, the frequency ratio method and PCA are used to eliminate the influence of regional characteristics factors and reduce the dimensions of the remaining influence factors. Finally, the SVM model is applied to assess landslide susceptibility under an assumed earthquake along the Dayong Highway in the Chenghai earthquake-prone area.

### PCA-based methods

PCA is a well-known multivariate analysis technique for reducing data dimensions^34^. It helps in reducing the data dimensionality by rotating coordinate axes. The PCA involves an eigenvalue decomposition to produce eigenvalues and eigenvectors for detecting the change range of data and transforming high dimensional data into low dimensional data. With the help of principal component analysis, a large number of sample data are replaced by a small number of principal components, which can not only maintain the classification of the original data but also reduce the dimension of features and eliminate the physical meaning of parameters, so as to facilitate more intuitive and effective classification. The variables obtained after dimensionality reduction contain most of the required information and avoid the interaction between variables. Mathematically, the reduction process is achieved by taking $p$ variables ${X}_{1},{X}_{2},\dots ,{X}_{p}$ which are then combined to produce principal components (PCs) ${PC}_{1},{PC}_{2},\dots ,{PC}_{p}$, that are uncorrelated. These PCs are also termed eigenvectors. The model between the principal components and the dependent variables is established after the extraction of the principal components.

### Support vector machine

**Figure 4:**
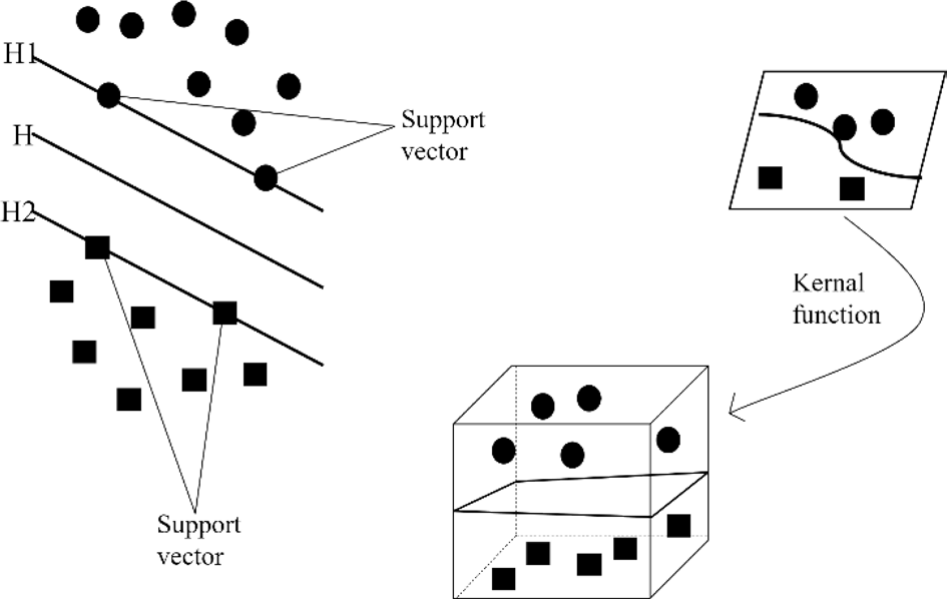
Illustrations of the basic principles of SVM.

The SVM is a commonly used machine learning algorithm that combines the Vapnik–Chervonenkis Dimension from statistics with Structure Risk Minimization Theory. It is widely used in decision-making and prediction in various fields, and can also classify and regress the data. The two main principles of SVM are the optimal classification hyperplane and the use of a kernel Function^35^. SVM can also manage linearly inseparable problems by utilizing current data for training and selecting several support vectors from training data to represent all data. Compared to both the logistic regression and neural networks, the support vector machine or the SVM sometimes gives a cleaner and sometimes more powerful way of learning complex nonlinear functions. Illustrations of the basic principles of SVM are shown in Fig. 4. By leading to a mapping function, the kernel function maps the sample feature attributes from low dimensional space to high dimensional space and then transforms the nonlinear classification problem into a linear classification problem in high dimensional space. The commonly used kernel functions include linear kernel function, polynomial kernel function, radial basis function kernel and sigmoid kernel function. According to the experience of some scholars in the application of landslide susceptibility classification, the Gaussian kernel function is selected for training which has the advantages of less training parameters and low complexity of the model. The calculation process is as follows Eq. (1)

1 $$K\left({x}_{1},{y}_{1}\right)={e}^{-y{\left({x}_{1}-{x}_{2}\right)}^{2}}$$

## Susceptibility assessment model of seismic landslides based on historical earthquake

### seismic landslides sample selection

**Figure 5:**
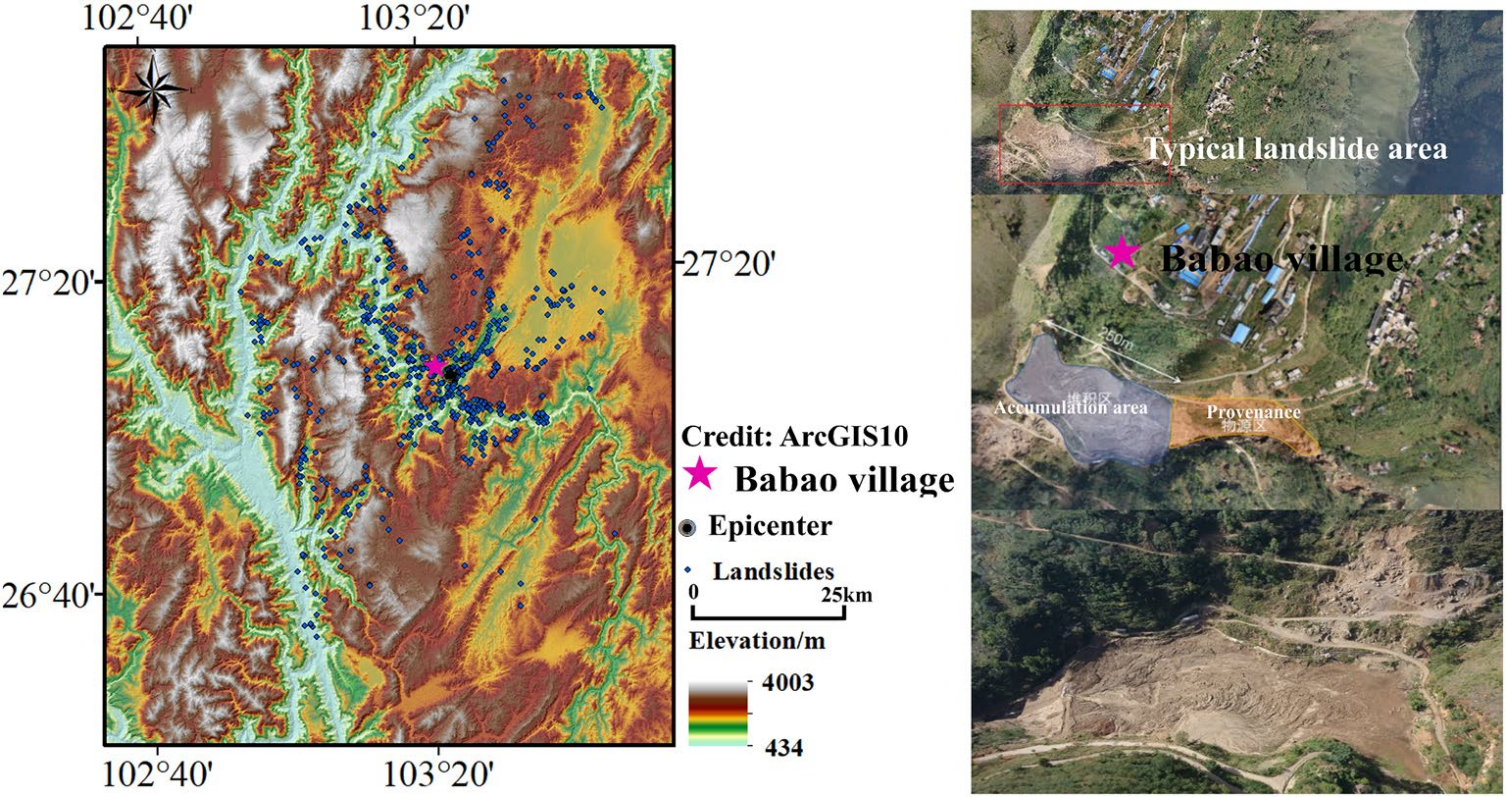
Landslide distribution of the Ludian area. (Credit: 1,2,5,6,11,12, ArcGIS10, URL: https://gisserver.domain.com:6443/arcgis/manager).

On August 3, 2014 (at 4:30 p.m.), the Ms6.5 earthquake occurred in Ludian County, Zhaotong City, Yunnan Province. The epicenter of the earthquake was located at 27.1° N, 103.3° E and the focal depth was 12 km. Conducted by the Earthquake Administrator in Yunnan Province, 716 new landslide points triggered by this earthquake were recognized through field surveys and UAV tilt photography. (Fig. 5). This damaging earthquake caused approximately 400 deaths, 1800 injuries, and the destruction of at least 12,000 houses.

### Selection of seismic landslide sample points

**Figure 6:**
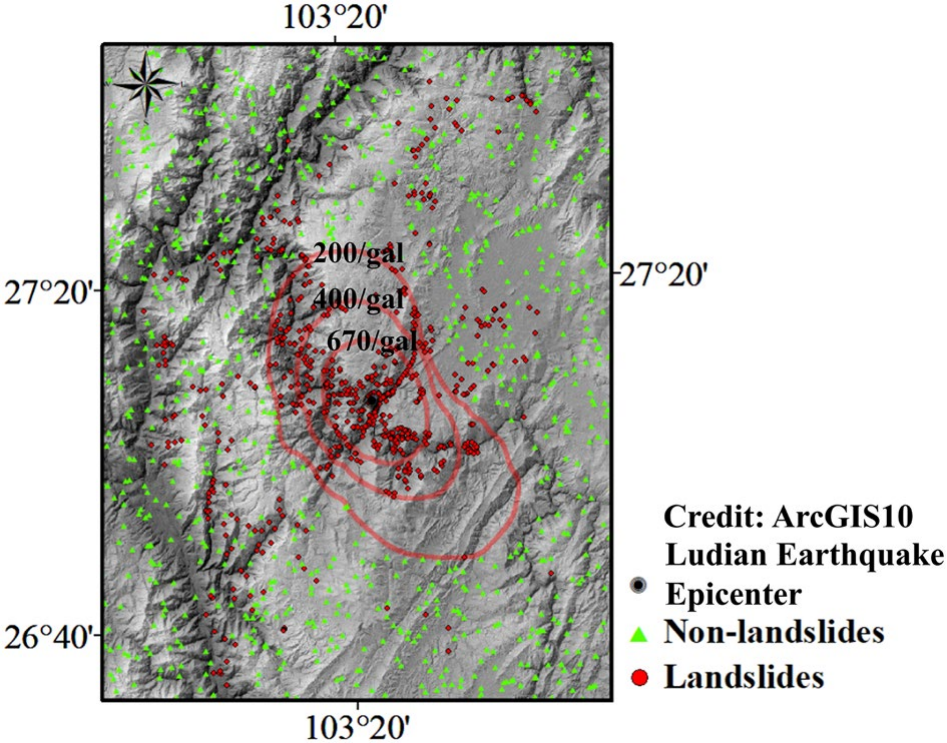
The landslides units and non-landslides units digital map in Ludian area. (Credit: 1,2,5,6,11,12, ArcGIS10, URL: https://gisserver.domain.com:6443/arcgis/manager).

In this paper, a DEM with a resolution of 30 × 30 m is adopted for the grid computing units of the study area. According to the landslide inventory map, there are 716 slope units containing the entire known landslide body. Landslide buffer zones are created around the landslide points. Specifically, circular buffer zones with a radius of 1000 m are generated around each landslide point as the center. The collection of all the circular buffer zones is called the landslide buffer zone. To meet the modelling requirements and improve the operation accuracy, the random sampling method is used to generate non-landslide points (2130) which is the 3× the number of landslide units outside the landslide buffer zone. The landslide and non-landslide units in the study area are shown in the Fig. 6.

### Landslide influence factors

Based on previous research, Landslides are triggered by various processes including geological, geomorphological, meteorological factors, human engineering activities, groundwater level coefficient and ground motion. There is no unified standard for the selection of assessment indexes. Landslides in the Ludian area are mostly affected by seismic activity. The factors influencing landslides can be divided into internal factors and external factors. In this study, the external incentive factors of seismic landslides mainly consider earthquake factors, including epicenter distance and PGA. In addition, eight factors including elevation, slope, slope direction, distance to stream, distance to fault, geology, terrain wetness index (TWI) and normalized difference vegetation index (NDVI) are selected as internal influencing factors. The selection and sources of indicators are shown in Table 1, and the elevation, slope, slope direction and terrain wetness index are calculated by DEM grid map in ArcGIS. The DEM data is from the geospatial data cloud and the accuracy is 30 m.

**Table 1:** Selection of assessment indexes.

First class	Second class	Third class	Data	Type
External factors	Seismic factor	Horizontal PGA	PGA records of seismic stations	Data sheet
		Epicenter distance	DEM	Raster map
Internal factors	topographic features	Elevation	DEM (30 m accuracy)	Raster map
		Slope		
		Aspect		
	Hydrogeology	Distance to stream		
		TWI		
	Geology	Geology	geological map (1:200,000 scale)	Vector image Raster map
		Distance to fault		
	Vegetation cover	NDVI	modis(16 day NDVI data, 250 m accuracy)	Raster map
Inventory of landslides	Landslide area distribution		Vector map of original landslide distribution	Vector image

### Model established and verification

Ten seismic landslide impact factors are normalized and entered the support vector machine. Equation (2) is implement to normalize the values of the impact factors of Ludian seismic landslide.

2 $${{x}{\prime}}_{i}=\frac{{x}_{i}-{x}_{min}}{{x}_{max}-{x}_{min}}$$

where ${x}_{i}{\prime}$ and ${x}_{i}$ indicate the normalized and original values of each impact factor, ${x}_{max}$ and ${\mathrm{x}}_{\mathrm{min}}$ indicate the maximum and minimum values of each impact factor. In the training process of SVM model, the main purpose is to determine the kernel function parameter $\gamma$ and penalty factor *C*. The five-fold cross-validation method is used to validate the models and to overcome the shortage of landslide data and the problem of model overfitting^36^. The calculation step is dividing all the landslide and non-landslide point data into 5 folds. Each fold is used in turn as the validation set, while the remaining folds are used as the training set. This process was repeated 5 times. By constantly changing the parameter values, the corresponding classification accuracy can be calculated, and then the classification accuracy can be used to determine the optimal parameter combination. Through the above training, we have obtained the optimal kernel function parameter $\gamma$ and penalty factor* C*.

Receiver operating characteristic curve (ROC) is often used as a quantitative analysis method to assessment the prediction accuracy of landslide sensitivity model. The abscissa of the curve is false positive rate (*FPR*), *N* is the number of real negative samples, and *FP* is the number of positive samples predicted by the classifier. The ordinate is true positive rate (*TPR*), *P* is the number of real positive samples, *TP* is the number of positive samples predicted by the classifier. The formula is as follows Eq. (3):

3 $$TPR=\frac{TP}{P}$$

Introduce the calculation formula of accuracy using Eq. (4), where *TN* is the true negative and *FN* is the false negative.

4 $$A\text{ccuracy} = \frac{\left(TP+TN\right)}{\left(TP+TN+FP+FN\right)}$$

**Figure 7:**
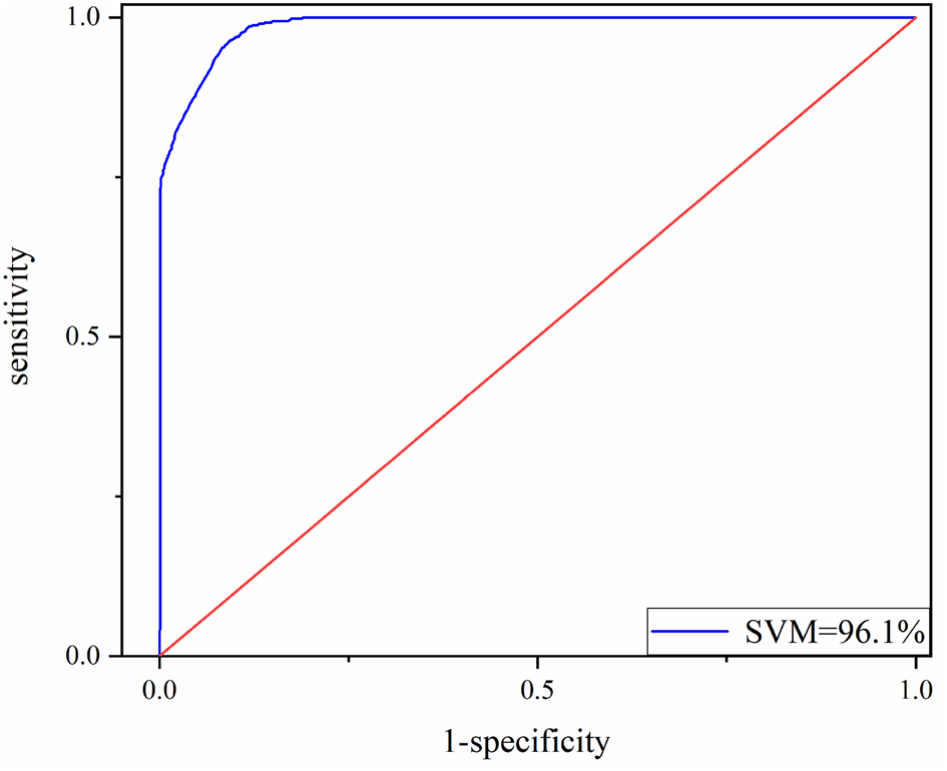
ROC curve of SVM model.

The area under the curve is called AUC (area under ROC curve), AUC is generally between 0.5 and 1, the larger AUC value indicates that the performance of the model is better. when $\gamma =0.8$, *C* = 0.5, the model accuracy is the highest, AUC = 96.1%. The ROC curve is as shown in the Fig. 7. The results demonstrate the accuracy and rationality of the assessment model in landslide prediction.

## Application in Chenghai earthquake-prone area

### Improvement of model universality

#### Screening of influencing factors

In order to reduce the dependence of the model on specific landslides data and increase model universality, FR method is used to screen the impact factors of seismic landslides. The calculation process is described in Eq. (5)

5 $$FR=\frac{L{/L}_{1}}{S/{S}_{1}}$$

**Figure 8:**
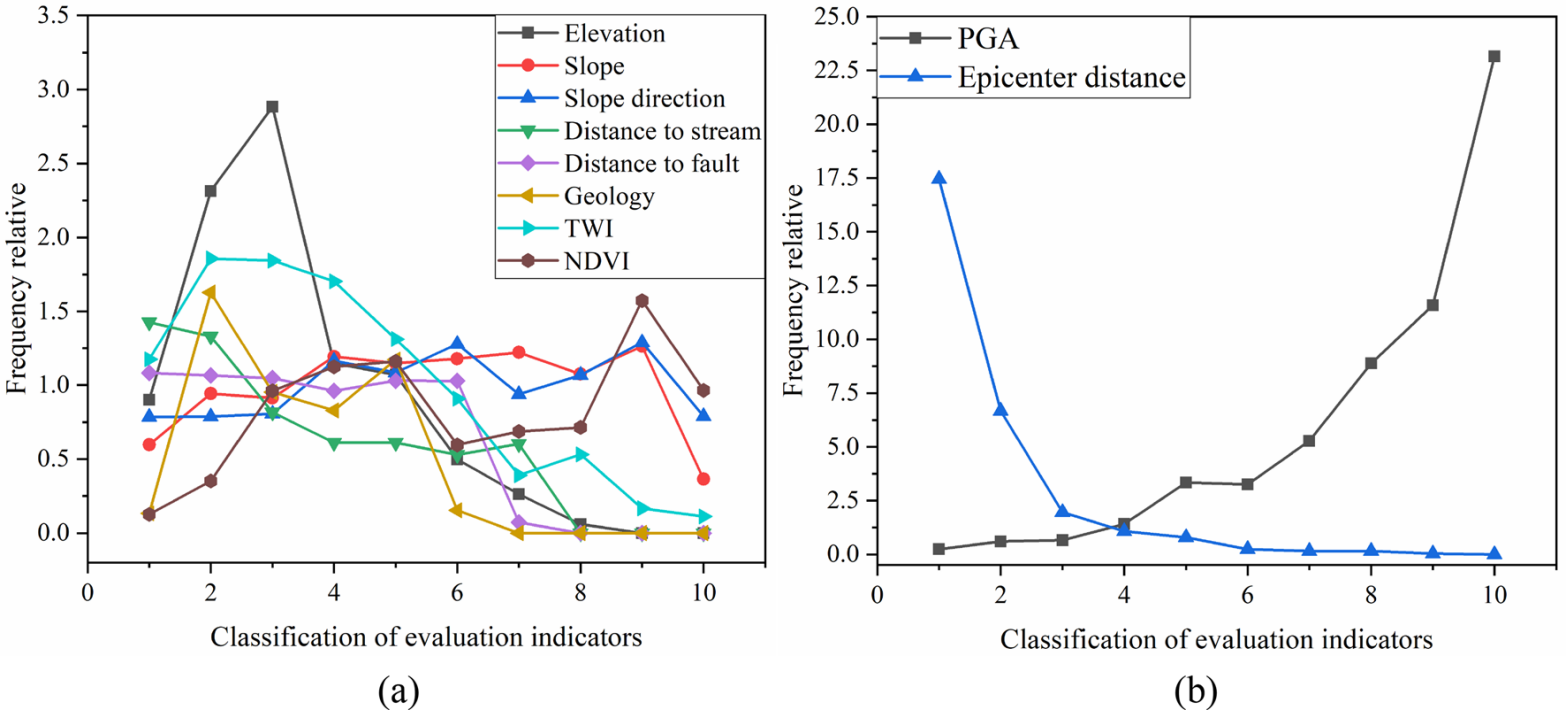
Frequency statistics of landslide influence factor. (**a**) Internal factors. (**b**) External factors.

$FR$ is frequency relative of landslide, $L$ is the number of landslide points in the classification, ${L}_{1}$ is total number of landslide points in research area. $S$ is the acreage of classified area, and ${S}_{1}$ is the total study area. The line chart of the frequency ratio of all influence factors is shown in the Fig. 8, from the frequency ratio results, the landslide frequency under the influence of elevation, geology, and TWI factors are concentrated in the local range. Therefore, the relationship between those factors and regional characteristics is relatively close. For other factors shown in Fig. 8a, the distribution of landslides frequency in this area is discrete. The relationship between landslide distribution and the external trigger factor of PGA and epicenter distance is shown in Fig. 8b, the curve indicates the frequency of landslides rising with the increase of PGA and epicenter distance. There is no doubt that PGA and epicenter distance are the main factors affecting seismic landslides. However, it can be concluded that elevation, geology, and TWI conditions can be defined as regional influence characters because of their direct linear relationship with the occurrence of earthquake-induced landslides. Therefore, those influence factors should be deleted to improve the generalization ability of the model in the seismic landslide susceptibility assessment of the Chenghai region.

#### Dimension reduction of influencing factors

The reasonable application of the seismic landslide susceptibility assessment model based on SVM in the Chenghai area requires good robustness. PCA method is used to extract feature vectors and reduce the dimensions of input variables. Through Spearman’s rank correlation coefficient, the linear correlation relationships of influence factors are summarized as follows in Table 2. It proves that there is a low linear correlation between the influencing factors. Meanwhile, the value for KMO is 0.602 and the degrees of freedom of Butler’s spherical test is 21. It is reasonable and feasible to extract the susceptibility assessment indexes of seismic landslides according to the PCA method.

**Table 2:** Component matrix in Ludian area.

Component matrix	Slope	Slope direction	Distance to stream	Distance to fault	NDVI	PGA	Epicenter distance
Slope	1						
Slope direction	0.023	1					
Distance to stream	0.089	− 0.021	1				
Distance to fault	0.089	− 0.021	0.385	1			
NDVI	0.18	− 0.014	0.103	0.103	1		
PGA	0.123	0.041	− 0.352	− 0.352	0.058	1	
Epicenter distance	0.126	− 0.03	0.127	0.127	0.1	0.689	1

**Figure 9:**
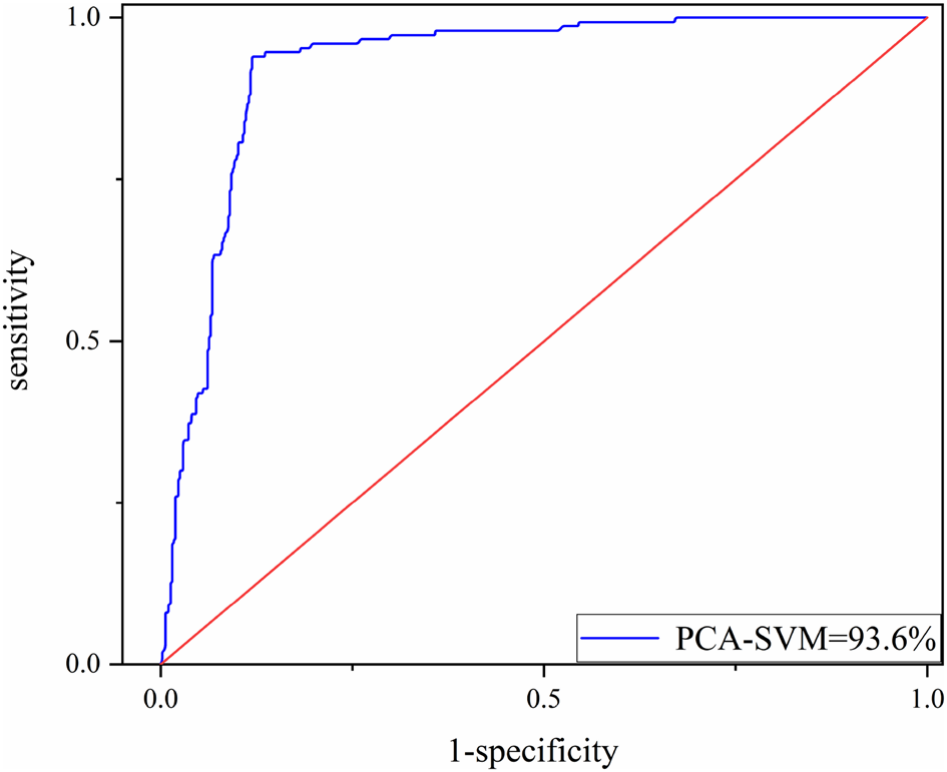
ROC curve of PCA–SVM model.

Table 3 shows, the five principal components represent 84.618% content of the impact factors. Generally, the external factors including PGA and epicenter distance have the most effect on principle components $P_{1}$ indicates more than 28.025% input variables variance proportions. It is reasonable to take the impact of the Ludian earthquake on landslides into consideration. Moreover, the slope has the most effect on principle components $P_{2}$, slope direction has the most effect on principle components $P_{3}$. $P_{4}$ principle component includes the distance to fault as the main effective factor. And the distance to stream is the main effective factor of the principle components $P_{5}$. By using the PCA method, the input slope, PGA and other influencing factors of the seismic landslide susceptibility assessment model are transformed into dimensionless principal components. The dependence of the model on original geographic information data and seismic data is reduced, and the robustness of the model is increased. The parameters of the PCA–SVM are consistent with those of the original SVM model, and the accuracy is the highest. Figure 9 shows, that after FR and PCA methods for data processing, the accuracy of the PCA–SVM is 93.6%. Compared with the original support vector machine model, the accuracy of the PCA–SVM model has not significantly decreased and maintained over 90%.

**Table 3:** Results of PCA method in Ludian area.

Principal components		P_1_	P_2_	P_3_	P_4_	P_5_
	Slope	− 0.159	0.74	0.232	0.13	0.556
	Slope direction	0.002	− 0.195	0.751	0.6	− 0.12
	Distance to stream	0.4	− 0.511	− 0.011	− 0.059	0.709
	Distance to fault	0.194	0.122	0.598	− 0.745	− 0.096
	NDVI	0.462	0.485	− 0.212	0.153	− 0.11
	PGA	− 0.877	− 0.028	− 0.078	− 0.027	0.034
	Epicenter distance	0.87	0.058	− 0.054	0.107	− 0.11
Contribution rates/%		28.025	15.714	14.714	13.864	12.301
Accumulative contribution rate/%		28.025	43.739	58.453	72.317	84.618

The coefficient of determination R2 is adopted for judging the fitting degree of models. The coefficient of determination R2 is calculated as:

$$R^{2} = 1 - \frac{{\sum {(y_{i} - \hat{y}_{i} )^{2} } }}{{\sum {(y_{i} - \overline{y})^{2} } }}$$

where $y$ is the actual label value, $\hat{y}$ is the predicted value, and $\overline{y}$ is the average of the actual values. The coefficient of determination ranges from 0 to 1. The closer the predicted value is to the actual label value, the smaller the error, and the closer the coefficient of determination is to 1. After calculation, the R^2^ of the original SVM model is 0.915, and the R^2^ of the PCA–SVM model is 0.875.

### Assuming an earthquake in the Chenghai area

**Figure 10:**
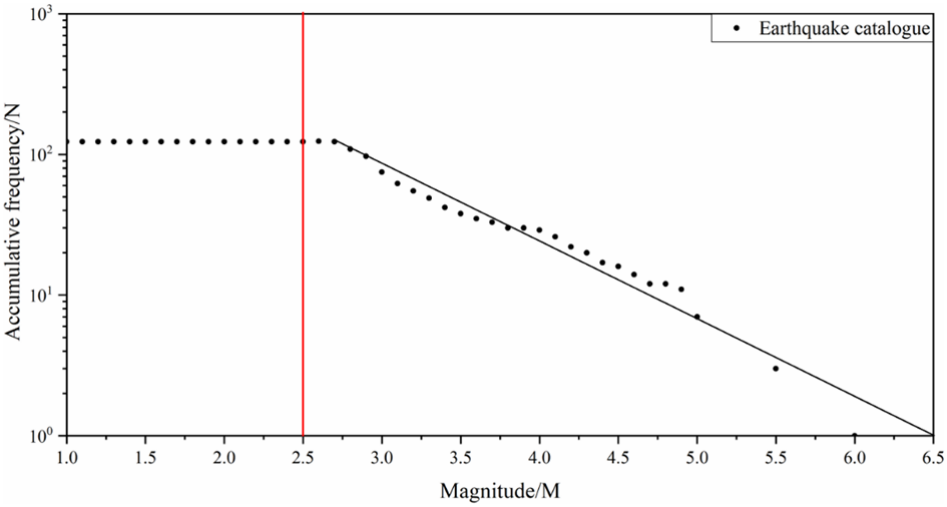
Magnitude-frequency curves.

To apply the SVM model for assessing the seismic landslide susceptibility of the Dayong highway, we assume that an earthquake caused by the Chenghai fault zone in the future. The G–R model is used to study the relationship between magnitude and frequency, and then the upper limite of magnitude is determined. The G-R model is expressed as Eq. (6).

6 $$logN\left(M\right)=a-b\times M$$

where $N\left(M\right)$ is the frequency of earthquakes with magnitude greater than or equal to $M$, $a$ and $b$ are parameters. The G–R model was used to fit the regional magnitude-frequency relationship. The seismic statistics are collected in the recent ten years (2011–2021) from the Earthquake Administration of Yunnan Province. The magnitude-frequency relation curve is shown in the Fig. 10. The data is complete and the linear relationship is well when the magnitude is between 2.5 and 6.0, this data is used in the G-R model for calculating the maximum magnitude in the Chenghai area.

According to the G-R model and the calculation rules of the least square method, the values of parameters $a$ and $b$ can be calculated,$a=4.362$, $b=0.671$. In general, the upper limited of magnitude is the maximum magnitude that can occur in the region, and the number of occurrences is once. Therefore, through assume the $N\left({M}_{max}\right)$=1, the ${M}_{max}$ is calculated. ${M}_{max}=6.5.$ Probability of occurrence of maximum magnitude is calculated based to the Dieterich model expressed as Eqs. (7) and (8).

7 $$R(t)=\frac{r}{\left[{e}^{\left(-\frac{\Delta CFS}{A\sigma }\right)}-1\right]{e}^{\left(-\frac{t}{{t}_{a}}\right)}+1}$$

8 $$P=1-{e}^{\left(-R\right)}$$

where $P$ represents the occurrence probability, $r$ is the frequency of the target seismic magnitude, which is 0.01, $\Delta CFS$ is the Coulomb stress, which is 0.024, $A\sigma$ = 0.1, and ${t}_{a}$ is assumed to be 10. According to the Dieterich model, the occurrence probability of an Ms6.5 earthquake induced by the Chenghai fault in the next 10 years is 1.1%. The epicenter can be located at 26.20° N and 100.60° E. This position is selected as a potential epicenter because it is the epicenter of the Yongsheng Ms 6.0 earthquake. Yongsheng Ms 6.0 earthquake is induced by the Chenghai fault, and seismicity is vigorous in this area where the latest Ms4.9 earth-quake occurred at 26.16° N and 100.62° E on July 21, 2019. We assume that the PGA distribution is the same as that of the Ludian earthquake, and obtain the PGA distribution of the Chenghai area by Kriging interpolation.

### Regional topographic and geological characteristics

**Figure 11:**
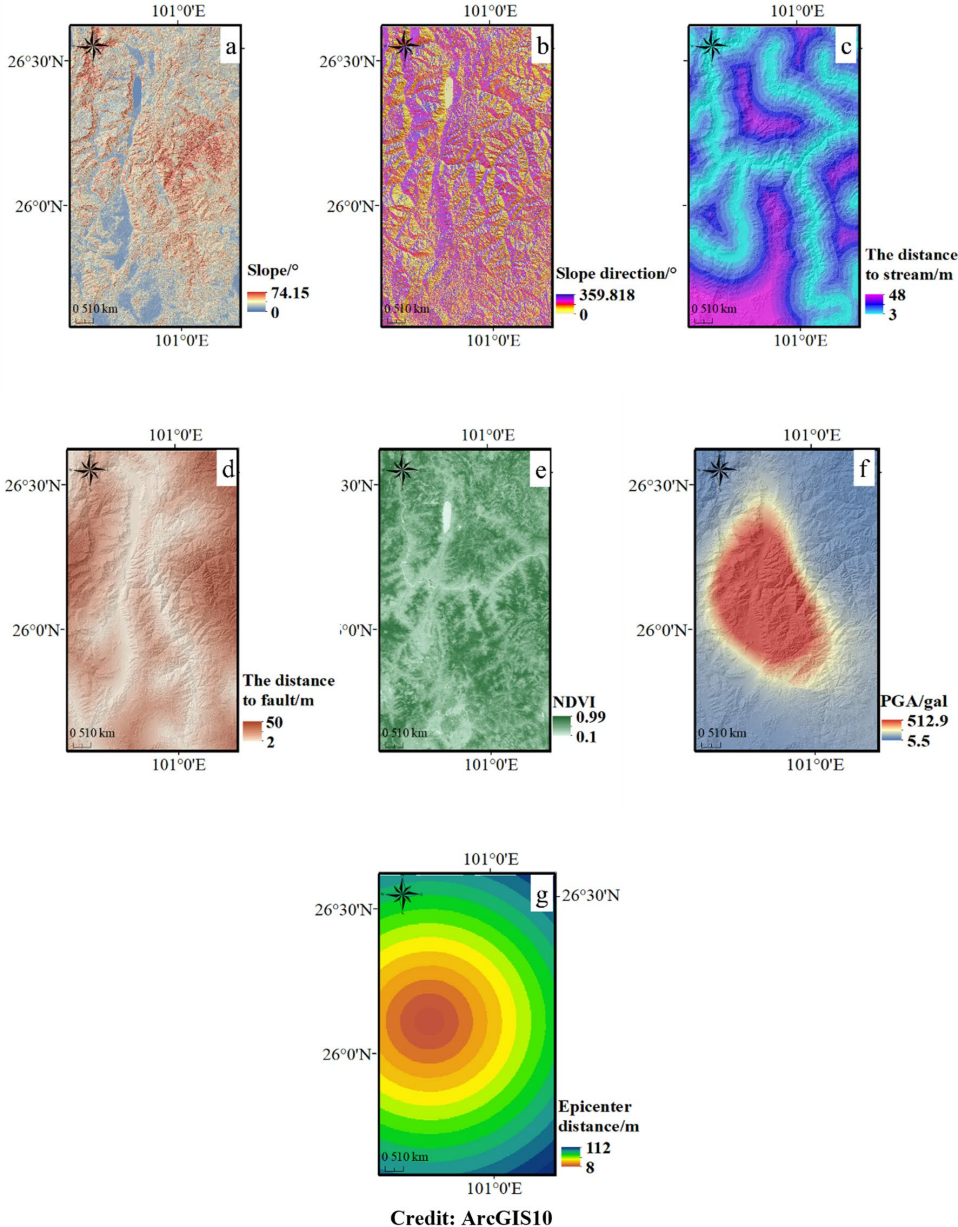
The influence factors digital map. (**a**) Slope, (**b**) Slope direction, (**c**) the distance to stream, (**d**) The distance to fault, (**e**) NDVI, f PGA, (**g**) Epicenter distance. (Credit: 1,2,5,6,11,12, ArcGIS10, URL: https://gisserver.domain.com:6443/arcgis/manager).

The topographic, geological, and ground motion digital map of the study area is obtained by ArcGIS with a spatial resolution of 30 m × 30 m (Fig. 10). The elevation ranges from 1000 to 4000 m, the slope is mostly between 10 and 40° and the terrain is higher in the northeast and northwest. The main strata in the area are Quaternary Holocene, Permian, Carboniferous, and Devonian, which consist of limestone and basalt. Generally, the topographic and geomorphological characteristics in this area are very similar to those in the Ludian area. Therefore, the rationality of applying the landslide susceptibility assessment model trained using the Ludian seismic landslide data to the Chenghai area is much higher than that of other regions. Moreover, the PCA–SVM model with improved robustness through FR and PCA methods can maintain high accuracy when applied to the Chenghai earthquake-prone region. The digital map of influence factors in the Chenghai area is shown in Fig. 11.

Through the PCA, five principal components were extracted from ten landslide factors in the Chenghai area, the five principal components are shown in Table 4. The five principal components represent 90% of the original data in the Chenghai region. By using the PCA method, generous seismic landslide influence factors data is dimension reduced. The factors that have the greatest impact on principal components in the Chenghai area are basically consistent with those in the Ludian area. It is profitable to prevent excessive deviation in the application process of the seismic landslide susceptibility assessment model.

**Table 4:** Component matrix in Chenghai area.

Principle components		P_1_	P_2_	P_3_	P_4_	P_5_
	Slope	0.228	0.875	0.035	− 0.347	0.074
	Slope direction	− 0.05	0.047	− 0.013	0.214	0.972
	Distance to stream	0.638	− 0.051	− 0.429	0.114	− 0.012
	Distance to fault	0.638	− 0.051	− 0.429	0.114	− 0.012
	NDVI	0.313	0.305	0.514	0.449	− 0.14
	PGA	− 0.874	0.457	− 0.32	0.289	− 0.089
	Epicenter distance	0.464	− 0.432	0.615	− 0.292	0.099
Contribution rates/%		27.228	21.488	17.161	13.867	11.955
Accumulative contribution rate/%		27.228	48.716	65.877	79.744	90.699

### Seismic landslide susceptibility mapping

**Figure 12:**
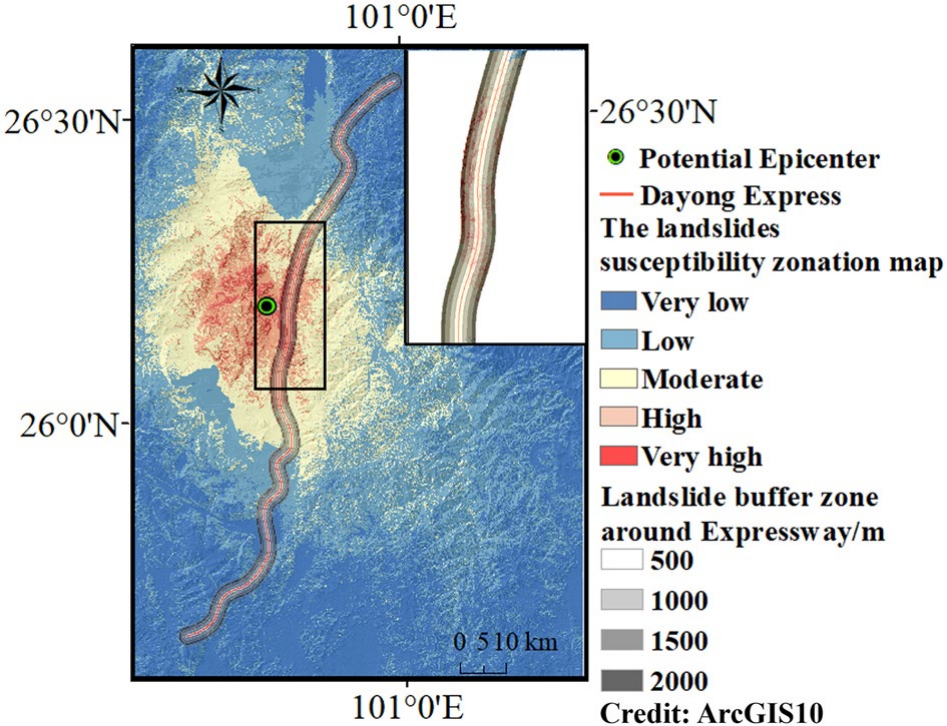
The seismic landslide susceptibility zoning map. (Credit: 1,2,5,6,11,12, ArcGIS10, URL: https://gisserver.domain.com:6443/arcgis/manager).

By inputting the dataset from the Chenghai area into the PCA–SVM model which is trained by using Ludian earthquake data. The landslide susceptibility zoning map of the Chenghai area is obtained. According to the classification of the landslide susceptibility by the natural break, the seismic landslide susceptibility values are classified into five classes (Very low, Low, Moderate, High, and Very high). This class is based on natural groupings inherent in the data and boundaries are determined statistically where there are relatively large jumps in the susceptibility data values^37^. The seismic landslide susceptibility zoning map is shown in Fig. 12. The “Very low” class area is represented by dark blue, and the “Very high” class area is represented by crimson. The colors changing from cold to warm shows that the susceptibility class of landslide increases gradually.

**Figure 13:**
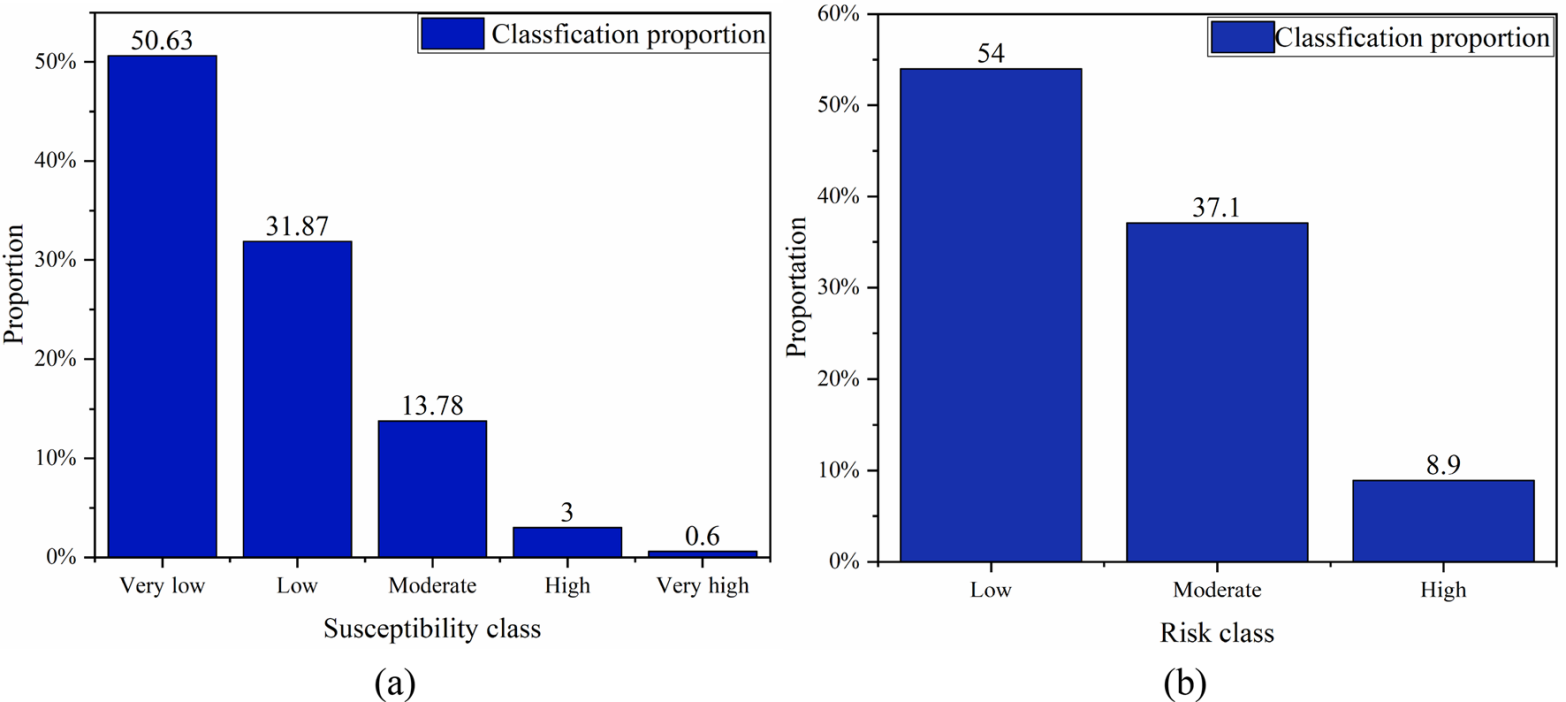
(**a**) Statistics of grid data of landslides susceptibility class of Chenghai area. (**b**) Statistics of grid data of risk class sections of Dayong Highway.

Figure 12 shows that the landslide susceptibility class distribution which is similar to the attenuation law of ground motion decreases gradually from the inside out around the epicenter. The landslide susceptibility mapping show that under the influence of hypothetical ground motions, the “Very high” areas are distributed in the range of 14.5 km around the earthquake center. The spatial distribution is concentrated on both sides of the epicenter. The “High” areas are distributed in the range of 20 km around the earthquake center. Different from the “Very high” areas, the “High” areas are mainly distributed within 20 km from the epicenter and are mostly concentrated on the outer side. The distribution range of the “Moderate” is basically consistent with the distribution range of the PGA and extends towards the northwest and southeast directions. As the distance from the epicenter continues to increase, the landslide susceptibility class declines. It is worth noting that there are still some areas with high landslide susceptibility class in areas over 25 km from the epicenter, the PGA of these areas is relatively small. This is due to its own internal reasons leading to a higher landslide susceptibility class, such as slope, distance to faults and distance to stream. Figure 13a shows the number of grid cells of the susceptibility class represented by a histogram. The “Low” and “Very Low” areas account for over 80% of the entire Chenghai region. “Moderate” or above accounting for 17.38% of the total Chenghai area. However, the “Very High” area accounts for only 0.6% of the total Chenghai region. According to the seismic landslide susceptibility mapping, the landslide susceptibility class is higher within a range of 15 km from the epicenter, and lower in other regions.

### Assessment results

According to the landslide susceptibility results in the Chenghai area, the susceptibility of landslides along different sections of the Dayong highway is assessed. The risk level of the Dayong highway is affected by both the seismic landslide susceptibility level and landslide travel distance. Based on statistics of the travel distances for 54 seismic landslides triggered by the 2008 Wenchuan earthquake^38^, most landslides travelled 0–2000 m. Therefore, the travel distance along the Dayong highway is divided into four parts from 0 to 2000 m. The resulting landslide susceptibility map and estimated seismic landslide travel distances provide the basis for the risk assessment of the Dayong highway. Table 5 illustrates a risk matrix used to generate the risk heatmap by integrating the landslide susceptibility results along the highway and landslide travel distances. Considering the seismic landslide susceptibility mapping and the landslide travel distance, the Inverse Distance Weight (IDW) method can be used for drawing the risk heatmap, which can visually show the risk ranking of the Dayong Highway. The IDW gridding method can be either an exact or a smoothing interpolator. With IDW, data are weighted during interpolation such that the influence of one point relative to another declines with distance from the grid node^39^. Approximately 35% of the highway passes through the “Very high” and “High” landslide susceptibility class area, mainly concentrated in the middle section of Dayong Highway. The coincidence length between the middle section of the Dayong Highway and the Chenghai fault zone is about 13 km. The nearest distance is only approximately 4.5 km away from the Dayong Highway, the earthquake center, which is greatly affected by ground motion and has the highest susceptibility class resulting from landslides. In the south and north of the middle section of the Dayong highway, a total of 34 km section is parallel to the Chenghai fault zone. This section passes through the “High” landslide susceptibility class area and with high susceptibility landslide-prone points nearby. Approximately 60% of the highway sections are in moderate and below moderate susceptibility areas.

**Table 5:** Risk matrix of Dayong Highway.

Landslide susceptibility	Landslide travel distance			
	0–500 m	500–1000 m	1000–1500 m	1500–2000 m
Very high	High	High	High	Moderate
High	High	High	Moderate	Low
Moderate	High	Moderate	Moderate	Low
Low	Moderate	Moderate	Low	Low
Very low	Moderate	Low	Low	Low

Based on the assessment principle, Fig. 13b shows the proportion of sections in Dayong Highway with different landslide risk classes. High-risk sections account for 8.9% of the total highway, which is most vulnerable to landslides. Moderate-risk sections account for 37.1%, and it is located on the north and south sides of high-risk sections. The low-risk sections are located on the north and south sides of Dayong Highway and have the largest proportion 54%.

**Figure 14:**
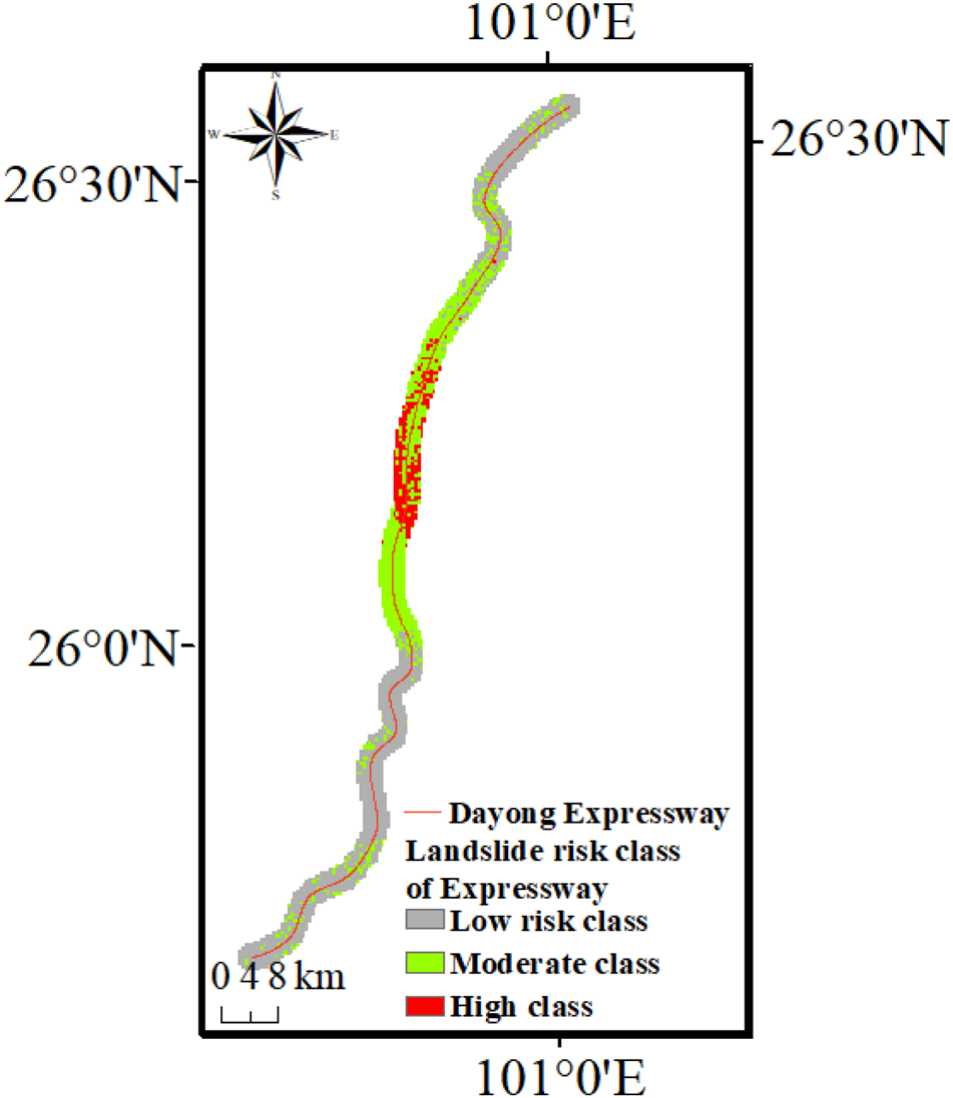
Landslide risk class of Dayong Highway.

Figure 14 shows the seismic landslide risk of the Dayong highway. The “High” risk class section is about 20 km and is concentrated in the middle part of the Dayong highway. Most of them are over 1000 m away from the Dayong highway and near the potential epicenter. The “Moderate” risk class section is distributed on the north and south sides of the “High” risk class section. The southern part is about 36 km, and the northern part is about 44 km. The “Low” risk class section is mainly located at the north and south ends of the Dayong Expressway. The north part is about 30 km, and the south part is about 70 km.

## Discussions

PCA is a linear dimensionality reduction method that can reduce the dimensionality of geological and environmental factors that affect landslides while minimizing information loss. This is beneficial for improving the robustness and applicability of machine learning models. As a data processing method, PCA has the following advantages: (1) effectively reducing the data dimensions of landslide impact factors and improving machine learning computational efficiency; (2) Being able to retain the main information in landslide impact factor data and reduce the difficulty of data processing. (3) It can eliminate the physical significance of landslide impact factor data and improve the robustness of machine learning. Hence, the PCA method can simplify the ML processes and improve their robustness.

This work used basic classifiers SVM for the landslide susceptibility map. Optimizing input data and parameters is a suitable method to exert the model's generalization performance. The SVM model performs well in fitting small sample size data. When the sample size is small, SVM can achieve good classification results. Based on the research findings^40^, we compared the performance of SVM and RF models in landslide susceptibility analysis under small sample nonlinear conditions. The accuracy of SVM is 0.998, and the accuracy of RF is 0.999, with only a 0.1% difference. Moreover, compared with the random forest method, the SVM model has strong generalization ability. It tends to maintain high accuracy to control model complexity and avoid overfitting. However, the random forest method is prone to overfitting training data. After PCA analysis, the influencing factor data was transformed into low dimensional nonlinear data, and the SVM model has more advantages in dealing with nonlinear binary classification problems and has a shorter training time than Random Forest and more stable results. The Random Forest results are affected by the decision tree growing process, and each run may be different. In this landslide susceptibility analysis, the sample size is small, and more attention is paid to generalization capability, so SVM is a better choice. But Random Forest also has advantages, such as better adaptability to nonlinear classification and high dimensional data. The choice needs to be made according to the specific situation.

This research illustrates that the PCA–SVM model performs better in application ability. Meanwhile, the decrease in prediction accuracy is not significant. SVM models have significant advantages when dealing with problems with small sample sizes. Some authors have specifically discussed the performance of SVM models in predicting landslide hazards and showed that the SVM model might derive a higher prediction accuracy than other models when dealing with binary classification and lack of data problems (Huang, Kamila, Miloš, Yao.). Our result is consistent with the conclusions of these authors.

The landslide susceptibility of the Chenghai area has also been modelled by other authors. One Tang et al. proposed a risk assessment method in the Chenghai area based on the fractal theory and the K-means cluster method. Compared with the previous works, the application ability of this work is greatly improved, and the seismic landslide mapping is basically consistent. Thus, the methodology proposed in this study is considered effective and extendable to other areas where geographic environment information is similar for landslide hazard mapping.

## Conclusions

1. In this study, 10 influencing factors including internal and external factors are selected as the landslide susceptibility assessment indexes based on the Ludian earthquake. Three impact factors including elevation, geology, and TWI with poor dispersion were removed through the frequency ratio method. The seven last seismic landslide influence factors are reduced to the five-principal components which represent 84.618% content by principal component analysis. The reduced index system is used as the input to improve the universal of the SVM model. The results show that the accuracy of the support vector machine is 93.6% through AUC.
2. The possibility of an earthquake in the Chenghai area is relatively large in the future after analyzing the historical earthquake activities. An assume earthquake occurred in the Chenghai area by use of the Gutenberg-Richter method. The PCA–SVM model is applied in the Chenghai area and the seismic landslide susceptibility mapping is obtained. The landslide susceptibility assessment is optimal for dividing into five classes (Very low, Low, Moderate, High, and Very high).
3. The landslide susceptibility assessment results in the Dayong highway region indicate that under an assumed earthquake, the area with the “Very high” landslide susceptibility accounts for 0.23% and seismic landslide has the most obvious impact on the middle section part of Dayong highway which is parallel to Chenghai fault zone. Approximately 54% of the highway sections are in moderate and the following landslide susceptibility areas.
4. The landslide buffer zone is established around the Dayong highway and divided into four parts from 0 to 2000 m. “Very high” landslide susceptibility prone points are concentrated in the buffer zone of 1500–2000 and more than 2000 m. “High” landslide susceptibility prone points are distributed in 500–1500 m buffer zones on both sides of the Dayong highway. Most of the landslide-prone points are “moderate”, and minute quantities are “High” concentrated in a 0–500 m buffer zone. The landslide risk class of Dayong highway is obtained based on the seismic landslide susceptibility assessment and landslide travel distance. High-risk sections account for 8.9%, moderate-risk sections account for 37.1%, and low-risk sections account for 54%. Considering the maximum magnitude and occurrence probability in the Chenghai area, the landslide risk of Dayong Highway can be borne.

Our findings open several research directions for improving the generation ability of the earthquake landslide susceptibility model. It is worth emphasizing that in an earthquake-prone area, seismic landslide susceptibility analysis is very important for the large infrastructure projects construction. The PCA–SVM model presented in this paper can conduct seismic landslide susceptibility analysis when landslide data in the region is lacking, in order to reduce the risk of landslide disasters and formulate further development strategies.

## Data Availability

The data that support the findings of this study are available on request from the corresponding author, [Ailan Che], upon reasonable request.
